# Methotrexate upregulates circadian transcriptional factors PAR bZIP to induce apoptosis on rheumatoid arthritis synovial fibroblasts

**DOI:** 10.1186/s13075-018-1552-9

**Published:** 2018-03-22

**Authors:** Kohjin Suzuki, Kohsuke Yoshida, Takeshi Ueha, Kenta Kaneshiro, Ayako Nakai, Naonori Hashimoto, Koto Uchida, Teppei Hashimoto, Yoshiko Kawasaki, Nao Shibanuma, Natsuko Nakagawa, Yoshitada Sakai, Akira Hashiramoto

**Affiliations:** 1 Department of Biophysics, Kobe University Graduate School of Health Sciences, Tomogaoka 7-10-2, Suma-ku, Kobe, 654-0142 Japan; 2 Division of Rehabilitation Medicine, Kobe University Graduate School of Medicine, Kobe, 650-0017 Japan; 3 Department of Rheumatology, Kobe Kaisei Hospital, Kobe, 657-0068 Japan; 4 Department of Orthopedic Surgery, Kobe Kaisei Hospital, Kobe, 657-0068 Japan; 5 Department of Rheumatology, Hyogo Prefectural Kakogawa Medical Center, Kakogawa, 675-0003 Japan

**Keywords:** Rheumatoid arthritis, Synovial fibroblasts, Methotrexate, Proline and acidic amino acid-rich basic leucine zipper, Period2, Bcl-2 interacting killer

## Abstract

**Background:**

Effects of methotrexate (MTX) on the proliferation of rheumatoid arthritis (RA) synovial fibroblasts are incompletely understood. We explored actions of MTX in view of circadian transcriptions of synovial fibroblasts.

**Methods:**

Under treatment with MTX, expression of core circadian clock genes, circadian transcriptional factor proline and acidic amino acid-rich basic leucine zipper (PAR bZIP), and proapoptotic molecule Bcl-2 interacting killer (Bik) was examined by real-time polymerase chain reaction. Protein expression of circadian clock gene PERIOD2 (PER2) and CYTOCHROME C was also examined by western blotting and ELISA. Promoter activities of *Per2* and *Bik* were measured by Luciferase assay. Expression of PER2, BIK, and CYTOCHROME C and morphological changes of the nucleus were observed by fluorescent immunostaining. Synovial fibroblasts were transfected with *Per2*/*Bik* small interfering RNA, and successively treated with MTX to determine cell viabilities. Finally, synovial fibroblasts were treated with MTX according to the oscillation of *Per2/Bik* expression.

**Results:**

MTX (10 nM) significantly decreased cell viabilities, but increased messenger RNA expression of *Per2, Bik*, and PAR ZIP including D site of the albumin promoter binding protein (*Dbp*), hepatic leukemia factor (*Hlf*), and thyrotroph embryonic factor (*Tef*). MTX also increased protein expression of PER2 and CYTOCHROME C, and promoter activities of *Per2* and *Bik via* D-box. Under fluorescent observations, expression of PER2, BIK, and CYTOCHROME C was increased in apoptotic cells. Cytotoxicity of MTX was attenuated by silencing of *Per2* and/or *Bik*, and revealed that MTX was significantly effective in situations where *Per2*/*Bik* expression was high.

**Conclusions:**

We present here novel unique action of MTX on synovial fibroblasts that upregulates PAR bZIP to transcribe *Per2* and *Bik*, resulting in apoptosis induction. MTX is important in modulating circadian environments to understand a new aspect of pathogenesis of RA.

**Electronic supplementary material:**

The online version of this article (10.1186/s13075-018-1552-9) contains supplementary material, which is available to authorized users.

## Background

Methotrexate (MTX) is a folic acid antagonist widely used as an anchor drug in treating various cancers [1, 2], as well as rheumatoid arthritis (RA) [3]. For cancer cells, MTX competitively inhibits dihydrofolate reductase (DHFR) to block purine and pyrimidine biosynthesis, and thus it inhibits DNA replication and cell proliferation. For RA, low-dose MTX shows anti-inflammatory effects by inducing extracellular adenosine, which binds to adenosine receptors [4]. Moreover, it has been reported that MTX induced apoptosis in synovial fibroblasts in both *in-vivo* and *in-vitro* experiments [5]. Although MTX induced apoptosis in synovial fibroblast within 24–48 h [6, 7], the precise mechanism of how MTX expresses antiproliferative effects on synovial fibroblasts remains incompletely understood [8].

RA is a chronic arthritis characterized by ‘tumor-like’ synovial cell growth [9]. Another remarkable feature of RA is the circadian variation of disease-related symptoms, such as morning stiffness, increased production of proinflammatory cytokines at night time, and peaked secretion of immunoglobulin (Ig) A/IgM types of rheumatoid factor in the morning [10–15]. Since these rheumatic symptoms possess a daily rhythm, we have previously shown that the action of the biological clock was significantly disturbed in the mouse model of collagen antibody-induced arthritis [16] and that tumor necrosis factor (TNF)-α significantly disturbed the oscillation of biological clocks of synovial fibroblasts [17]. Fibroblasts usually demonstrate daily rhythms of circadian clock, while Haas *et al.* [18] pointed out that the expression rhythm of clock genes disappears in RA synovial fibroblasts presumably due to prolonged inflammation.

The circadian rhythm in human cells is mainly regulated by the core clock genes, including circadian locomotor output cycles kaput (*Clock*), brain and muscle Rant-like protein-1 (*Bmal1*), period (*Per*), and cryptochrome (*Cry*) [19–22]. The circadian transcriptional factor proline and acidic amino acid-rich basic leucine zipper (PAR bZIP) includes the D site of the albumin promoter binding protein (*Dbp*), hepatic leukemia factor (*Hlf*), and thyrotroph embryonic factor (*Tef*). PAR bZIP regulates gene expression by binding to a consensus sequence of D-box (5′-TTAXGTAA-3′; X = T or C) on the promoter region [23–25]. It has been reported that PAR bZIP can regulate the transcription of *Per2* gene by binding two D-box sequences existing on its promoter (D-box 1, 5′-TTATGTAA-3′, −372 to −365; and D-box 2, 5′-TTACGTAA-3′, −47 to −40) [24]. In contrast, E4-binding protein 4 (*E4bp4*) also binds to the D-box to suppress the transcription of *Per2* [26–28] (see Additional file 1).

It is noted that Bcl-2 interacting killer (Bik) possesses D-box (5′-TTAAGTCA-3′, −285 to −277) on its promoter region [28], resembling the arrangement of *Per2* genes. Bik, a member of the BH3-only subfamily, acts as an important signaling molecule upstream of the Bcl-2 and Bax subfamily [29]. The BCL-2 family has been known to be central players in regulating physiological activities of mitochondria, with Bcl-2, Bcl-xL, and Mcl-1 suppressing mitochondria-related apoptosis, whereas Bax and Bak induce it. Among these, Bik directly binds to these family proteins to induce apoptosis [29, 30].

In this study, we explored novel pharmacological effects of MTX on circadian clock genes and apoptosis induction in RA synovial fibroblasts.

## Methods

### Synovial fibroblast culture

Synovial tissues were obtained from RA patients (eight females and two males; aged 59.3 ± 3.4 years) during joint surgery. Eight of 10 patients took methotrexate and their average dosage was 7.4 ± 1.4 mg/week (Table 1) (see Additional file 2). This study has been approved by the ethics committee of Kobe University Graduate School of Health Sciences (#579–1) and Kobe Kaisei Hospital (#0072), in accordance with the Declaration of Helsinki. Written informed consent was obtained from each patient before study enrolment. Tissues were minced and treated with 2 mg/ml collagenase (Wako, Tokyo, Japan) in serum-free Dulbecco’s modified Eagle’s medium (DMEM; Nissui, Tokyo, Japan) at 37 °C for 1 h. Primary cultured synovial cell lines were established and maintained in DMEM including 10% heat-inactivated fetal bovine serum (FBS; Thermo, Waltham, MA, USA), 1% penicillin–streptomycin (100 U/ml; Life Technologies, Carlsbad, CA, USA), and 1% l-glutamine (2 mM; Life Technologies), in a humidified incubator at 37 °C in the presence of 5% CO_2_. Harvested cells were continuously cultured to obtain synovial fibroblasts, and cells of passages 3–6 were used in the entire experiments.

**Table 1:** Characterization of patients

Characteristic	Value
Patients (*n*)	10
Age (years)	59.3 ± 3.4
Sex (female/male)	8/2
Disease duration (years)	22.7 ± 3.2
C-reactive protein (mg/dl)	1.2 ± 0.4
DAS28-ESR	2.6 ± 0.4
Methotrexate (mg/week)	7.4 ± 1.4
Methylprednisolone (mg)	3.7 ± 0.9
Other DMARDs	
Adalimumab	2/10
Tocilizumab	2/10
Tacrolimus	2/10 (2, 1.5 mg)
Etanercept	2/10
Infliximab	1/10
None	2/10

### Synchronization of synovial fibroblasts by serum shock

Synovial fibroblasts were incubated in 50% horse serum and incubated for 2 h to synchronize the expression of clock genes [17, 31].

### Cell viability assay

Synovial fibroblasts (3.0 × 10^3^ cells) were cultured in serum-free DMEM with or without MTX (1, 10, and 100 nM, 1 μM; Sigma Aldrich, St. Louis, MO, USA). After incubating for 24–72 h, cell viabilities were measured as the 450-nm absorbance of reduced WST-8 (2-(2-methoxy-4-nitrophenyl)-3-(4-nitriphenyl)–5-(2, 4-disulphonyl)-2H–tetrazolium, monosodium salt) using the Cell Counting Kit-8 (Dojindo, Kumamoto, Japan). The values were represented relative to MTX-untreated cells (control).

### Real-time polymerase chain reaction

Synovial fibroblasts (8.0 × 10^4^ cells) were cultured in serum-free DMEM with or without MTX (10, 100 nM) for 24–32 h. After incubation, total RNA was extracted using the RNeasy Mini Kit (QIAGEN, Hilden, Germany). Then, reverse transcription was performed with the ReverTra Ace® qPCR RT Kit (Toyobo, Osaka, Japan) and analyzed on the StepOnePlus™ Real-Time PCR System (Applied Biosystems, Foster City, CA, USA). The TaqMan probes used were: Hs00154147_m1 for *Bmal1*, Hs00231857_m1 for *Clock*, Hs00256143_m1 for *Per2*, Hs00172734_m1 for *Cry1*, Hs00609747_m1 for *Dbp*, Hs00171406_m1 for *Hlf*, Hs01115720_m1 for *Tef*, Hs00993282_m1 for *E4bp4*, Hs00154189_m1 for *Bik*, and Hs00427621_m1 for TATA box binding protein (*Tbp*). Expression levels were normalized to *Tbp.*

### Western blotting

Synovial fibroblasts (5.0 × 10^5^ cells) were cultured in serum-free DMEM with or without MTX (10, 100 nM) for 24–48 h, and lysed with RIPA buffer (Wako) to obtain cytoplasmic proteins. Samples were subjected to sodium dodecyl sulfate polyacrylamide gel electrophoresis (SDS-PAGE), transferred to polyvinylidene difluoride (PVDF) membrane (Millipore, Bedford, MA, USA), probed with antibodies, and developed by ImmunoStar® LD (Wako). Antibodies (Abs) used were: anti-PER2 Ab (sc-101,105; Santa Cruz, Dallas, TX, USA), anti-CYTOCHROME C Ab (ab13575; Abcam, Cambridge, UK), anti-BIK Ab (NB100–56109; Novus, Littleton, CO, USA), anti-Actin Ab (sc-1616; Santa Cruz), anti-mouse IgG Ab (NA9310; GE Healthcare, Chicago, IL, USA), and anti-rabbit IgG Ab (NA9340; GE Healthcare).

### ELISA for PER2 and CYTOCHROME C

Synovial fibroblasts (3.0 × 10^3^ cells) were cultured in serum-free DMEM with or without MTX (10, 100 nM) for 32–48 h, and protein expression of PER2 and CYTOCHROME C was measured by the In Cell ELISA test kit (3440–02; Thermo). Antibodies were as already described.

### Luciferase reporter gene construction

Genomic DNA was obtained from RA synovial fibroblasts and amplified by PCR to generate the luciferase reporters. The primer pairs used for amplifying human *Bik* promoter containing D-box (−780 to +176) were 5′-TGGCCTAACTGGCCGTAAACAAGCTTTGCCGTGC-3′ (forward) and 5′-CGCCGAGGCCAGATCATGCTGGCAGCGTCTGTA-3′ (reverse). The primer pairs used for amplifying human *Bik* promoter without D-box (−260 to + 323) were 5′-TGGCCTAACTGGCCGCCTCTTGGAGCCTCGGTT-3′ (forward) and 5′-CGCCGAGGCCAGATCTTGCTGGAGCGGTAAAACC-3′ (reverse). The *KpnI* recognition sequence was added to the 5′ ends of forward primers and the *BglII* recognition sequence was added to the 5′ ends of reverse primers. The PCR products were cloned into the *KpnI* and *BglII* site of the pGL4.10 (luc2) vector (Promega, Madison, WI, USA) using In-Fusion® HD Cloning Plus (Takara, Shiga, Japan). The luciferase reporters containing *Per2* promoters were generated with reference to Yoshida *et al.* [17]. D-box motifs of *Per2* promoter were mutated from 5′-TTATGTAA-3′ to 5’-CGCCAGGC-3′ (−372 to −365) and from 5′-TTACGTAA-3′ to 5′-CAGCGTAA-3′ (−47 to −40) (see Additional file 3).

### Transient transfection and luciferase reporter assay

Synovial fibroblasts (4 × 10^4^ cells) were transfected with 500 ng of the pGL4.10 (luc2) vector containing various *Per2* and *Bik* promoters using Lipofectamine 3000 Transfection Reagent (Thermo). As an internal control, 35 ng of pRL-TK (Promega) containing the herpes simplex virus thymidine kinase promoter driving Renilla luciferase was cotransfected. After 24 h of incubation for transfection, cells were treated with 10 and 100 nM MTX for 24 h, and analyzed for luciferase activity using the Dual-Luciferase Reporter Assay (Promega). Activities of both firefly and Renilla luciferases were measured, and the activity of firefly luciferase was normalized by Renilla luciferase. The values were shown as relative variations to MTX-untreated cells.

### Fluorescent immunostaining

Synovial fibroblasts (6.0 × 10^3^ cells) were cultured in serum-free DMEM with or without MTX (10 nM) for 24 h. Then, cells were fixed with 4% formaldehyde, stained with anti-PER2 Ab, anti-BIK Ab, anti-CYTOCHROME C Ab, anti-mouse IgG (H + L), F(ab′)_2_ Fragment (Alexa Fluor® 594 Conjugate) (#8890; Cell Signaling Technology, Danvers, MA, USA), anti-rabbit IgG (H + L), F(ab′)_2_ Fragment (Alexa Fluor® 488 Conjugate) (#4412; Cell Signaling Technology), and DAPI (0.5 μg/ml; Sigma, St. Louis, MO, USA). Protein expression and morphological changes of the nucleus were examined under fluorescence microscopy.

### RNA interference

*Per2* small interfering (si) RNA (s16931; Life Technologies) and *Bik* siRNA (s1990; Life Technologies) were transfected into synovial fibroblasts (3.0 × 10^3^ cells) using Lipofectamine™ RNAiMAX (Life Technologies) for 48 h. Noncoding siRNA (4,390,843; Life Technologies) was also used as controls. After that, cells were cultured in serum-free DMEM with 10 nM of MTX for 24 h to measure cell viabilities using the Cell Counting Kit-8. The viabilities were represented relative to those of noncoding siRNA without MTX treatment.

### Statistical analyses

In dynamite-plunger plots, values were expressed as the mean ± standard error of the mean (SEM). In boxplots, values were expressed with 10th, 25th, 50th (median), 75th, and 90th percentiles, and the single values were superimposed on the boxplots using black symbols.

For statistical analyses, a one-sample Kolmogorov–Smirnov test was used to test the normality, A one-sample *t* test was used to compare one single control value and others, a paired *t* test was used to compare differences between two experimental groups, the Tukey test was used to compare differences between more than three experimental groups, and Dunnett’s test was used to compare differences between the control and others. All statistical tests were two-sided and *p* < 0.05 was considered statistically significant.

The statistical analyses were performed by EZR, based on R and R commander [32].

## Results

### MTX inhibited viabilities of synovial fibroblasts

**Fig. 1:**
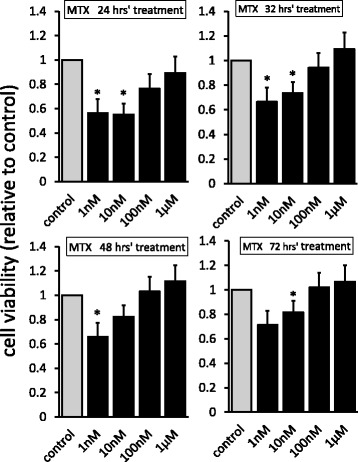
Cell viabilities of MTX-treated synovial fibroblasts. Cells treated with MTX (1, 10, and 100 nM, and 1 μM) or control media for 24–72 h. Cell viabilities of MTX-untreated cells defined as 1.0, and each value shown relative to control. Values are mean ± SEM. *p < 0.05 *vs* control, *n* = 5. MTX methotrexate

We first examined the effect of MTX on viabilities of synovial fibroblasts. A lower concentration of MTX (1, 10 nM) significantly decreased cell viabilities, while cell viability was relatively weak with a higher concentration of MTX (100 nM, 1 μM) (Fig. 1). Since 10 or 100 nM was the optimum MTX concentration in human sera [33], experiments were subsequently conducted with MTX 10 nM treatment as an appropriate effective concentration and a concentration of 100 nM as a less-effective control.

### MTX accelerated the expression of circadian clock gene *Per2*

**Fig. 2:**
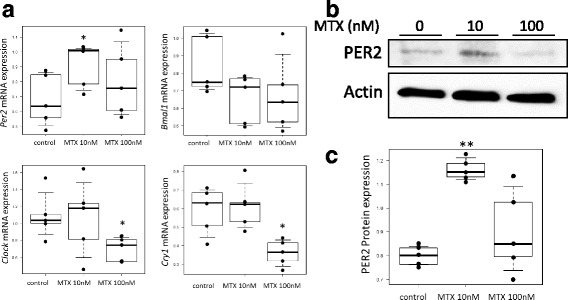
Effect of MTX on expression of core circadian clock genes in synovial fibroblasts. **a** Cells treated with MTX (10, 100 nM) or control media for 24 h to measure mRNA expression of *Per2, Bmal1, Clock,* and *Cry1*. Values given with 10th, 25th, 50th (median), 75th, and 90th percentiles and single values superimposed on the boxplots using black symbols. *p < 0.05 *vs* control, *n* = 5. **b** Cells treated with MTX (10, 100 nM) or control media for 32 h to observe PER2 expression. **c** Cells treated with MTX (10, 100 nM) or control media for 32 h to measure PER2 expression by ELISA. Values given with 10th, 25th, 50th (median), 75th, and 90th percentile and single values superimposed on the boxplots using black symbols. **p < 0.01 vs control, *n* = 5. Bmal1 brain and muscle Arnt-like protein-1, Clock circadian locomotor output cycles kaput, Cry cryptochrome, mRNA messenger RNA, MTX methotrexate, Per period

We next determined the effect of MTX on expressions of circadian clock genes. Under treatment with MTX (10 nM for 24 h), the expression of *Per2* messenger RNA (mRNA) was significantly increased, while expression of *Bmal1, Clock,* and *Cry1* mRNA was not. In contrast, under treatment with MTX (100 nM for 24 h), the expression of *Per2* did not show any significant differences, while the expression of *Clock* and *Cry1* significantly decreased (Fig. 2a). In accordance with this, the expression of PER2 protein was significantly increased by 10 nM of MTX for 32 h, but not by 100 nM (Fig. 2b, c).

### MTX increased the expression of PAR bZIP and *Bik*

**Fig. 3:**
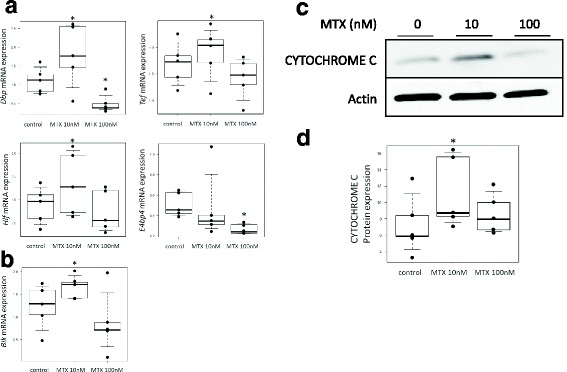
Effect of MTX on expression of circadian transcriptional factors PAR bZIP and *Bik* in synovial fibroblasts. **a** Cells treated with MTX (10, 100 nM) or control media for 24 h to measure expression of PAR bZIP gene mRNA (*Dbp, Tef,* and *Hlf*) and *E4bp4*. Values given with 10th, 25th, 50th (median), 75th, and 90th percentiles and single values superimposed on boxplots using black symbols. *p < 0.05 *vs* control, *n* = 5. **b** Cells treated with MTX (10, 100 nM) or control media for 24 h to measure expression of *Bik* gene mRNA. Values given with 10th, 25th, 50th (median), 75th, and 90th percentiles and single values superimposed on boxplots using black symbols. *p < 0.05 *vs* control, *n* = 5. **c** Cells treated with MTX (10, 100 nM) or control media for 48 h to observe CYTOCHROME C expression. **d** Cells treated with MTX (10, 100 nM) or control media for 48 h to measure CYTOCHROME C expression by ELISA. Values given with 10th, 25th, 50th (median), 75th, and 90th percentiles and single values superimposed on boxplots using black symbols. *p < 0.05 vs control, *n* = 5. Bik Bcl-2 interacting killer, Dbp D site of the albumin promoter binding protein, E4bp4 E4-binding protein 4, Hlf hepatic leukemia factor, mRNA messenger RNA, MTX methotrexate, Tef thyrotroph embryonic factor

The circadian transcriptional factor PAR bZIP (*Dbp, Tef*, and *Hlf*) and *E4bp4* genes regulate *Per2* gene expression by binding to D-box on the promoter region [23–28]. We therefore examined the effect of MTX on the mRNA expression of *Dbp, Tef, Hlf*, and *E4bp4*. Expression of *Dbp, Tef,* and *Tef* mRNA was significantly increased by 10 nM MTX treatment for 24 h, while *E4bp4* mRNA expression was not. However, expression of *Dbp* and *E4bp4* significantly decreased with 100 nM of MTX treatment (Fig. 3a).

Next, we examined the mRNA expression of the proapoptotic factor *Bik,* since the PAR bZIP-binding site also exists on the promoter region of *Bik* [29]. As shown in Fig. 3b, the expression of *Bik* mRNA was significantly increased by 10 nM of MTX treatment for 32 h.

Finally, we observed cytosolic release of CYTOCHROME C since Bik interacted with BCL-2 family proteins to induce mitochondria-related apoptosis. As a result, expression of CYTOCHROME C was increased by 10 nM of MTX treatment for 48 h, but not by 100 nM (Fig. 3c, d).

### Transcription of *Per2* and *Bik* was enhanced *via* D-box

**Fig. 4:**
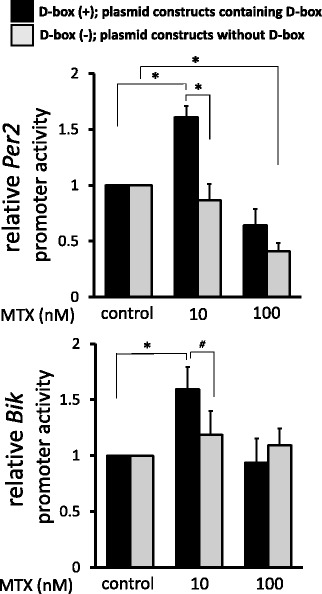
Promoter activities of *Per2* and *Bik* under MTX treatment. Synovial fibroblasts transfected with vectors containing various *Per2* or *Bik* promoters for 24 h. After treatment with MTX (10, 100 nM) or control media for 24 h, luciferase reporter assay was performed. Promoter activities of MTX-untreated cells defined as 1.0, and each value shown relative to control. Values are mean ± SEM. *p < 0.05, #*p* < 0.1, *n* = 5 (*Per2*), *n* = 6 (*Bik*). Bik Bcl-2 interacting killer, MTX methotrexate, Per period

To examine the roles of PAR bZIP in the transcription of *Per2* and *Bik*, we transfected luciferase reporters, constructed with or without D-box*,* to synovial fibroblasts. With 10 nM of MTX treatment for 24 h, D-box(+) *Per2* promoter activity was significantly increased as compared to those of the control and D-box(−) *Per2* promoter. By 100 nM of MTX treatment, D-box(−) *Per2* promoter activity was significantly decreased as compared to that of the control. *Bik* promoter activity was significantly increased after 10 nM of MTX treatment, and was higher than that of D-box(−) *Bik* promoter (Fig. 4).

### PER2, BIK, and CYTOCHROME C were highly expressed in apoptotic cells

**Fig. 5:**
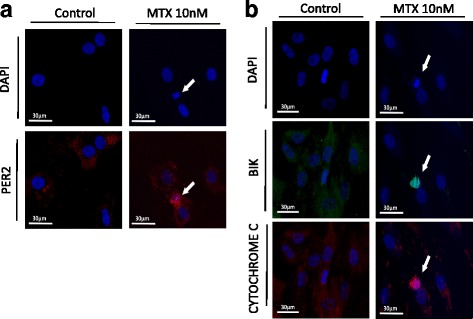
PER2, BIK, and CYTOCHROME C expression and morphological changes of the nucleus. **a** Cells stained with DAPI and anti-PER2 Ab after treatment with MTX (10, 100 nM) or control media for 24 h. Arrowheads indicate apoptotic cells. **b** Cells stained with DAPI, anti-BIK Ab, and anti-CYTOCHROME C Ab after treatment with MTX (10, 100 nM) or control media for 24 h. Arrowheads indicate apoptotic cells. Bik Bcl-2 interacting killer, DAPI 4′,6-diamidino-2-phenylindole, MTX methotrexate, Per period

To certify the specific expression of *Per2* and *Bik* on MTX-induced apoptotic synovial fibroblasts, we used fluorescent immunostaining to examine expressions of PER2, BIK, and CYTOCHROME C and, simultaneously, morphological changes of nucleus. As a result, nuclear blebbing, shrinkage, or fragmentation were observed indicating that MTX (10 nM for 24 h) could induce cell death. PER2 was overexpressed on apoptotic cells, but not on cells with normal appearance (Fig. 5a). BIK and CYTOCHROME C were also overexpressed specifically on the apoptotic cells (Fig. 5b).

### *Per2* and *Bik* are critical genes for MTX-induced apoptosis

**Fig. 6:**
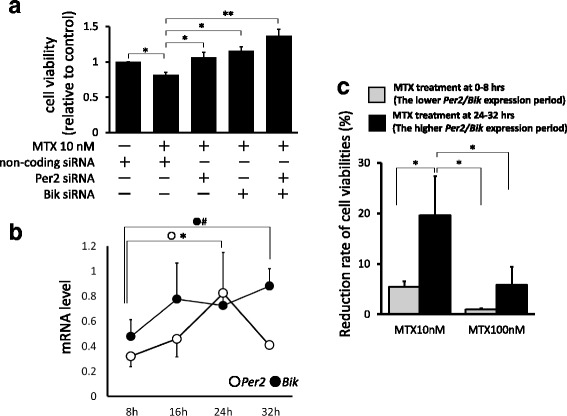
Cell viabilities after RNA interfering of *Per2* and *Bik,* and long-term expression of *Per2* and *Bik* mRNA under MTX treatment. **a** After transfection with *Per2* or *Bik* siRNA, cells treated with MTX (10, 100 nM) or control media for 24 h to measure cell viabilities. Cell viabilities of control (MTX-untreated cells) defined as 1.0, and each value shown relative to control. Values are mean ± SEM. *p < 0.05, **p < 0.01, *n* = 5. **b** Long-term expression of *Per2* and *Bik*. After 8, 16, 24, and 32 h of culture in serum-free media, mRNA was extracted from synovial fibroblasts to measure expressions of *Per2* and *Bik*. Values are mean ± SEM. *p < 0.05, #*p* < 0.1, *n* = 5. **c** Reduction rate of cell viabilities under time-dependent MTX treatments. Viabilities of synovial fibroblasts after treatment with 10 nM of MTX at 0–8 h (lower *Per2/Bik* expression period) or 24–32 h (higher *Per2/Bik* expression period) measured, respectively. Cell viabilities of MTX-untreated cells defined as 100%, and values are mean ± SEM. *p < 0.05, *n* = 5. Bik Bcl-2 interacting killer, mRNA messenger RNA, MTX methotrexate, Per period

To confirm whether PER2 and BIK were responsible for MTX-induced cell death, *Per2* and *Bik* siRNA were transfected into synovial fibroblasts to examine cell viabilities. As shown in Fig. 6a, cytotoxicity of MTX was attenuated by silencing of *Per2* and *Bik*, independently and synergistically.

When we examined the long-term expression of *Per2* and *Bik* mRNA (Fig. 6b), they gradually increased from 8 h until 24 or 32 h after synchronized circadian oscillations by serum shock, consistent with our previous report that the expression of *Per2* on synovial fibroblasts was increased after synchronization and peaked at 24 h [17]. Since these genes were responsible for MTX-induced cell death, synovial fibroblasts were separately treated by 10 nM of MTX at 0–8 h (the lower *Per2/Bik* expression period) and at 24–32 h (the higher *Per2/Bik* expression period), and viabilities of synovial fibroblasts were again measured. As shown in Fig. 6c, the reduction rate of cell viabilities was significantly increased by 24–32 h of MTX treatment compared with 0–8 h of treatment, depending on the expression levels of *Per2* and *Bik*.

## Discussion

We present here a novel pharmacological action of MTX in the viewpoint of circadian clock genes, circadian transcriptional factor PAR bZIP, and proapoptotic molecule Bik. In addition, a crucial mechanism of MTX-induced apoptosis on rheumatoid synovial fibroblasts was further traced.

In this study, the effect of MTX on cell viabilities did not show a concentration-dependent manner, with a wide range of MTX concentrations from 1 nM to 1 μM. In addition to our result, we found that MTX concentrations lower than 1 nM did not decrease cell viability (see Additional file 4). MTX is transferred almost equally to synovial fluid and sera, and the concentration of MTX in human sera can reach 200 nM at the peak and immediately decreases to less than 10 nM [33], although pharmacological effects on cytotoxicity or cellular viability may not necessarily increase in a concentration-dependent manner. As reported previously, viabilities of human lymphoblastic leukemia cells and epithelial cells of rat decreased in a concentration-dependent manner at low MTX concentrations, but did not show a significant decrease at high MTX concentrations [34, 35]. Moreover, MTX did not affect viability in a concentration-dependent manner on T cells and RA synovial fibroblasts, in contrast to those of dose dependency observed in osteoarthritis synovial fibroblasts [6, 36]. Thus, further studies are required for the action of MTX correlated with drug dosage or cell types.

Since MTX concentrations below 100 nM were conceivable in sera of RA patients, we next focused on the effect of MTX on circadian clock genes and circadian transcriptional factor PAR bZIP genes and their relations to mitochondria-related apoptosis of synovial fibroblasts.

Since circadian clock genes were reported to be closely related to the pathogenesis of arthritis [16, 37] and excessive expression of *Per2* could induce apoptosis [38], we examined the effect of MTX on expression of *Per2*, *Bmal1*, *Clock,* and *Cry1* that were regarded as “core” clock genes [20–22]. We examined mRNA expression of circadian clock genes over time, and found that the controls and 10/100 nM of MTX showed almost the same expression rhythms, and MTX influenced their expression levels (see Additional file 5).

As described, both *Per2* and *Bik* genes have D-box in their promoter regions, and PAR bZIP proteins regulate the expression of these genes by binding to D-box [24, 39]. Indeed, promoter activities of *Per2* and *Bik* were upregulated by 10 nM of MTX when the D-box sequence exists and were cancelled without D-box. Moreover, both PER2 and BIK were highly expressed in MTX-induced apoptotic cells, while inhibitions of *Per2* and *Bik* synergistically attenuated the effect of MTX on cellular viabilities. As it has been reported that PAR bZIP and Bik mediate oxidative stress-induced apoptosis in fibroblasts [39], we consider *Per2* and *Bik* as essential factors for MTX-dependent synovial cell death and propose here that two independent pathways can mediate these death signals: the PAR bZIP–*Per2* transcriptional pathway and the PAR bZIP–*Bik* transcriptional pathway (see Additional file 6). However, as shown in Fig. 2a, downregulation of *Clock* and *Cry1* appeared to be less effective on cell viabilities after treatment with 100 nM of MTX. Further *in-vivo* study should be required for clinical application by investigating the cross-talk of clock genes.

Last, because of the similarity of their promoter region containing D-box, we supposed *Bik* might show oscillation as has been reported for *Per2* [17, 31]. For the further understanding of circadian manifestation in rheumatoid arthritis, we showed that the expression of *Bik*, as well as *Per2*, was gradually increased from 8 h until 24 or 32 h, and MTX was significantly effective in situations when *Per2* and *Bik* were highly expressed. It has been reported that administration of MTX at bedtime, as an optimal dosing time associated with the oscillation of TNF-α production, could reduce the disease activities of patients with RA [40, 41]. Thus, we considered that expression levels of *Per2* and *Bik* could also be a critical biomarker for chronotherapy of MTX not only for RA, but also for other diseases such as acute lymphocytic leukemia, non-Hodgkin’s lymphoma, osteosarcoma, and breast cancer, in accordance with a previous report that circadian clock genes could be a biomarker for chronotherapy [42].

## Conclusions

We propose here that MTX induces apoptosis in synovial fibroblasts through the binding of PAR bZIP to D-box of two different genes, *Per2 and Bik*, and these dual pathways work independently but synergistically. In consequence, MTX acts as a modulator of circadian environments of synovial fibroblasts in relation to biological clock genes and circadian transcriptional factors.

## Additional files

Additional file 1: The interactions of circadian clock genes and its relative factors. BMAL/CLOCK and PER/CRY create the circadian rhythm via E-box, and DBP, TEF, HLF, E4BP4 via D-box. BMAL and CLOCK heterodimerize and bind to E-box elements on promoter regions of Per and Cry genes to induce their transcription. Thereafter, PER and CRY proteins heterodimerize to inhibit activities of their own and other E-box regulated promoters. (PDF 214 kb)

Additional file 2: Detailed information of patients with RA enrolled in this study. Joint samples obtained from 10 different patients to establish primary cultured synovial fibroblast cell lines. Age, sex, disease duration, CRP, DAS28-ESR, and treatment with MTX, PSL, and other DMARDs shown in the table. (PDF 252 kb)

Additional file 3: Plasmid constructs of *Per2/Bik* promoter. D-box(+), plasmid constructs containing D-box. D-box(−), plasmid constructs without D-box. D-box motifs of Per2 promoter were mutated from 5′-TTATGTAA-3′ to 5′-CGCCAGGC-3′ (−372 to −365), and 5′-TTACGTAA-3′ to 5′-CAGCGTAA-3′ (−47 to −40). Human Bik promoter containing D-box (−780 to +176) and human Bik promoter without D-box (−260 to +323) constructed. (PDF 215 kb)

Additional file 4: Cell viability of MTX-treated fibroblasts. Cell viability of RA synovial fibroblasts measured by WST-8 assay after 24 h of stimulation of MTX (1 pM to 1 μM). MTX (1 and 10 nM) significantly decreased cell viability as shown in Fig. 1, while 1–100 pM of MTX did not. (PDF 274 kb)

Additional file 5: The mRNA expression of circadian clock genes over time. mRNA expression of circadian clock genes measured at –4 h (before synchronization), 0 h (just before MTX stimulation), 24 h, 32 h, and 48 h. Controls and 10/100 nM of MTX showed almost the same expression rhythms, and MTX influenced their expression levels. (PDF 264 kb)

Additional file 6: Two different transcriptional pathways by which MTX induces apoptosis to synovial fibroblasts: PAR bZIP–*Per2* transcriptional pathway and PAR bZIP–*Bik* transcriptional pathway. We propose MTX induces apoptosis in synovial cells through activated binding of PAR bZIP to D-box in two different genes, *Per2* and *Bik*, and these dual pathways work independently but synergistically. (PDF 206 kb)

## Abbreviations

Ab: Antibody
Bik: Bcl-2 interacting killer
Bmal1: Brain and muscle Arnt-like protein-1
Clock: Circadian locomotor output cycles kaput
Cry: Cryptochrome
Dbp: D site of the albumin promoter binding protein
DMEM: Dulbecco’s modified Eagle’s medium
E4bp4: E4-binding protein 4
FBS: Fetal bovine serum
Hlf: Hepatic leukemia factor
Ig: Immunoglobulin
mRNA: Messenger RNA
MTX: Methotrexate
PAR bZIP: Proline and acidic amino acid-rich basic leucine zipper
PCR: Polymerase chain reaction
Per: Period
PVDF: Polyvinylidene difluoride
RA: Rheumatoid arthritis
SDS-PAGE: Sodium dodecyl sulfate polyacrylamide gel electrophoresis
SEM: Standard error of the mean
siRNA: Small interfering RNA
Tbp: TATA box binding protein
Tef: Thyrotroph embryonic factor
TNF: Tumor necrosis factor
WST-8: 2-(2-Methoxy-4-nitrophenyl)-3-(4-nitrophenyl)-5-(2,4-disulfophenyl)-2H–tetrazolium
