# Supplementary material for: Methotrexate upregulates circadian transcriptional factors PAR bZIP to induce apoptosis on rheumatoid arthritis synovial fibroblasts
Source: Arthritis Res Ther. 2018 Mar 22;20:55. doi: 10.1186/s13075-018-1552-9 (PMC5863822; doi:10.1186/s13075-018-1552-9)
Supplement: Supplementary file 1 — The interactions of circadian clock genes and its relative factors. BMAL/CLOCK and PER/CRY create the circadian rhythm via E-box, and DBP, TEF, HLF, E4BP4 via D-box. BMAL and CLOCK heterodimerize and bind to E-box elements on promoter regions of Per and Cry genes to induce their transcription. Thereafter, PER and CRY proteins heterodimerize to inhibit activities of their own and other E-box regulated promoters. (PDF 214 kb) [file 13075_2018_1552_MOESM1_ESM.pdf]

Additional file 1

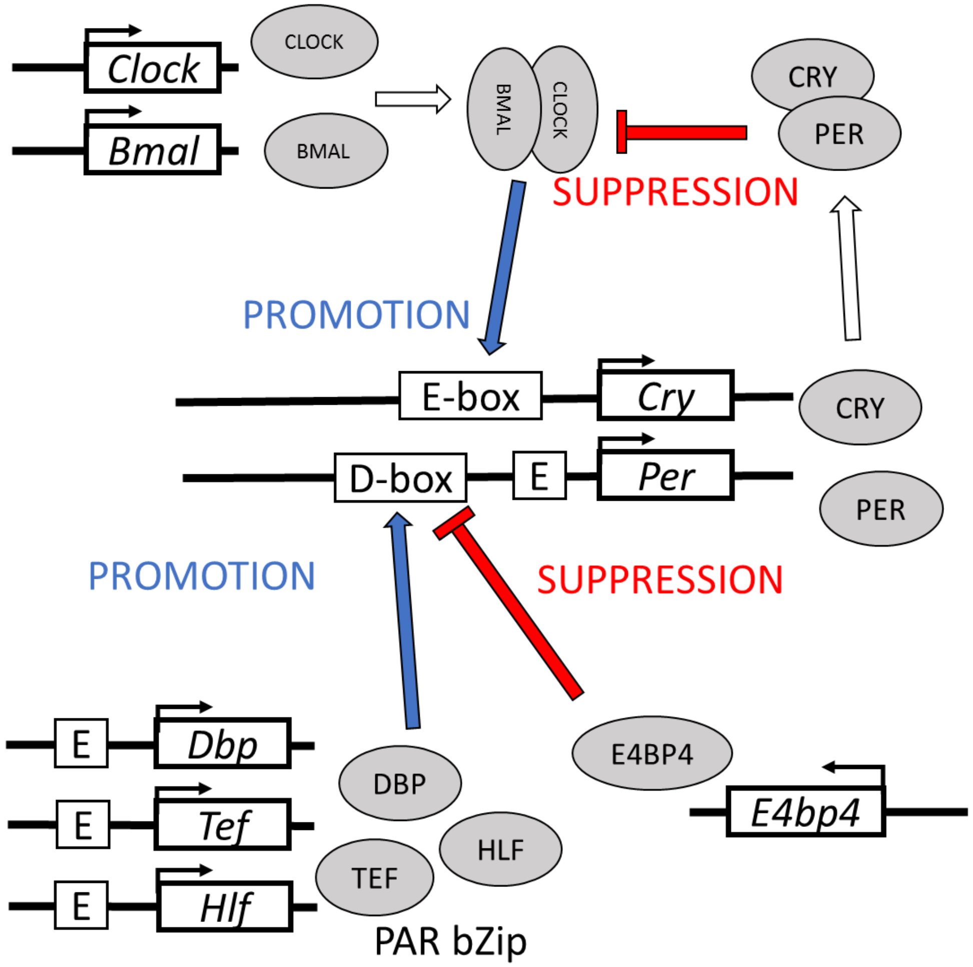

Additional file 1：

The interactions of circadian clock genes and its relative factors. BMAL/CLOCK and PER/CRY create the circadian rhythm *via* E-box, and DBP, TEF, HLF, E4BP4 *via* D-box.

BMAL and CLOCK heterodimerize and bind to the E-box elements on the promoter regions of *Per* and *Cry* genes to induce their transcription. Thereafter, PER and CRY proteins heterodimerize to inhibit the activities of their own and other E-box regulated promoters.
