# Supplementary material for: Methotrexate upregulates circadian transcriptional factors PAR bZIP to induce apoptosis on rheumatoid arthritis synovial fibroblasts
Source: Arthritis Res Ther. 2018 Mar 22;20:55. doi: 10.1186/s13075-018-1552-9 (PMC5863822; doi:10.1186/s13075-018-1552-9)
Supplement: Supplementary file 2 — Detailed information of patients with RA enrolled in this study. Joint samples obtained from 10 different patients to establish primary cultured synovial fibroblast cell lines. Age, sex, disease duration, CRP, DAS28-ESR, and treatment with MTX, PSL, and other DMARDs shown in the table. (PDF 252 kb) [file 13075_2018_1552_MOESM2_ESM.pdf]

Additional file 2

Additional Table

	age	sex	disease duration (yrs)	CRP (mg/dl)	DAS28-ESR	MTX (mg/w)	PSL (mg)	other DMARDs
Patient #1	56	F	19	2.08	3.8	12	3	none
Patient #2	67	F	32	1.1	2.7	6	0	ADA
Patient #3	62	F	21	0.02	0.6	0	10	Tac 2mg, TCZ
Patient #4	72	F	24	3.51	3.9	8	5	IFX
Patient #5	55	F	12	1.1	2.4	10	3	Tac 1.5mg
Patient #6	62	M	26	0.26	1.3	6	0	ADA
Patient #7	43	F	8	2.76	4.6	12	5	ETN
Patient #8	41	F	15	0.86	2.5	8	2.5	ETN
Patient #9	63	F	28	0.11	2.7	0	5	TCZ
Patient #10	72	M	42	0.32	1.6	12	3	none

Additional file 2 :

Detailed information of patients with RA, enrolled in this study.

Joint samples were obtained from 10 different patients to establish primary cultured synovial fibroblast cell lines.

Age, sex, disease duration, CRP, DAS28-ESR, and treatments with MTX, PSL, and other DMARDs were shown in the additional table.

MTX: Methotrexate, PSL: Methylprednisolone, DMARDs: disease modified anti-rheumatic drugs, ADA: Adalimumab, TCZ: Tocilizumab, Tac: Tacrolimus, ETN: Etanercept, IFX: Infliximab, ADA: Adalimumab.
