# Supplementary material for: Methotrexate upregulates circadian transcriptional factors PAR bZIP to induce apoptosis on rheumatoid arthritis synovial fibroblasts
Source: Arthritis Res Ther. 2018 Mar 22;20:55. doi: 10.1186/s13075-018-1552-9 (PMC5863822; doi:10.1186/s13075-018-1552-9)
Supplement: Supplementary file 3 — Plasmid constructs of Per2/Bik promoter. D-box(+), plasmid constructs containing D-box. D-box(−), plasmid constructs without D-box. D-box motifs of Per2 promoter were mutated from 5′-TTATGTAA-3′ to 5′-CGCCAGGC-3′ (−372 to −365), and 5′-TTACGTAA-3′ to 5′-CAGCGTAA-3′ (−47 to −40). Human Bik promoter containing D-box (−780 to +176) and human Bik promoter without D-box (−260 to +323) constructed. (PDF 215 kb) [file 13075_2018_1552_MOESM3_ESM.pdf]

Additional file 3

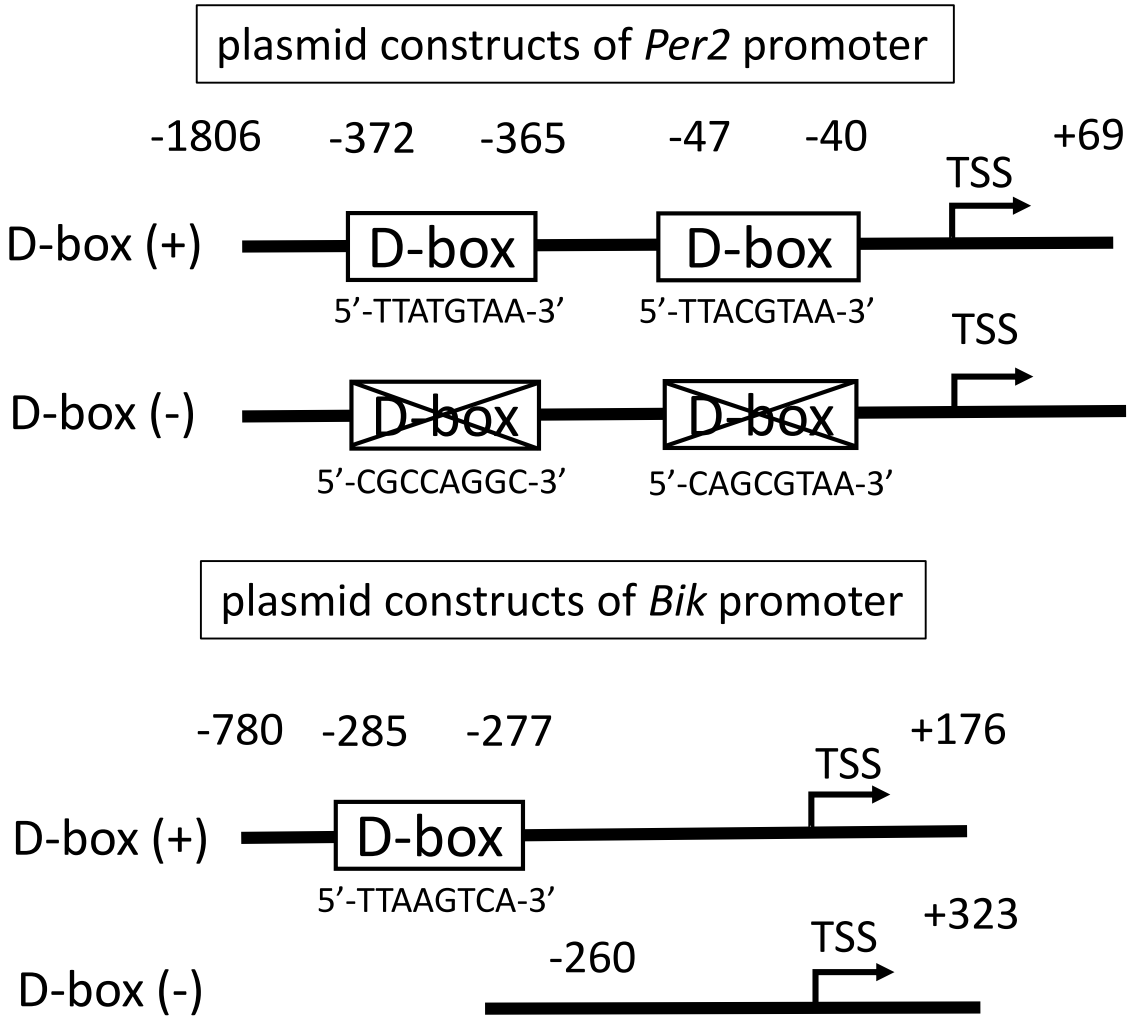

Additional file 3 :

D-box (+); plasmid constructs containing D-box. D-box (-); plasmid constructs without D-box.
D-box motifs of *Per2* promoter were mutated from 5’-TTATGTAA-3’ to 5’-CGCCAGGC-3’ (-372 to -365), and 5’-TTACGTAA-3’ to 5’-CAGCGTAA-3’ (-47 to -40).
Human *Bik* promoter containing D-box (-780 to +176) and human *Bik* promoter without D-box (-260 to +323) were constructed.
