# Supplementary material for: Methotrexate upregulates circadian transcriptional factors PAR bZIP to induce apoptosis on rheumatoid arthritis synovial fibroblasts
Source: Arthritis Res Ther. 2018 Mar 22;20:55. doi: 10.1186/s13075-018-1552-9 (PMC5863822; doi:10.1186/s13075-018-1552-9)
Supplement: Supplementary file 4 — Cell viability of MTX-treated fibroblasts. Cell viability of RA synovial fibroblasts measured by WST-8 assay after 24 h of stimulation of MTX (1 pM to 1 μM). MTX (1 and 10 nM) significantly decreased cell viability as shown in Fig. 1, while 1–100 pM of MTX did not. (PDF 274 kb) [file 13075_2018_1552_MOESM4_ESM.pdf]

Additional file 4

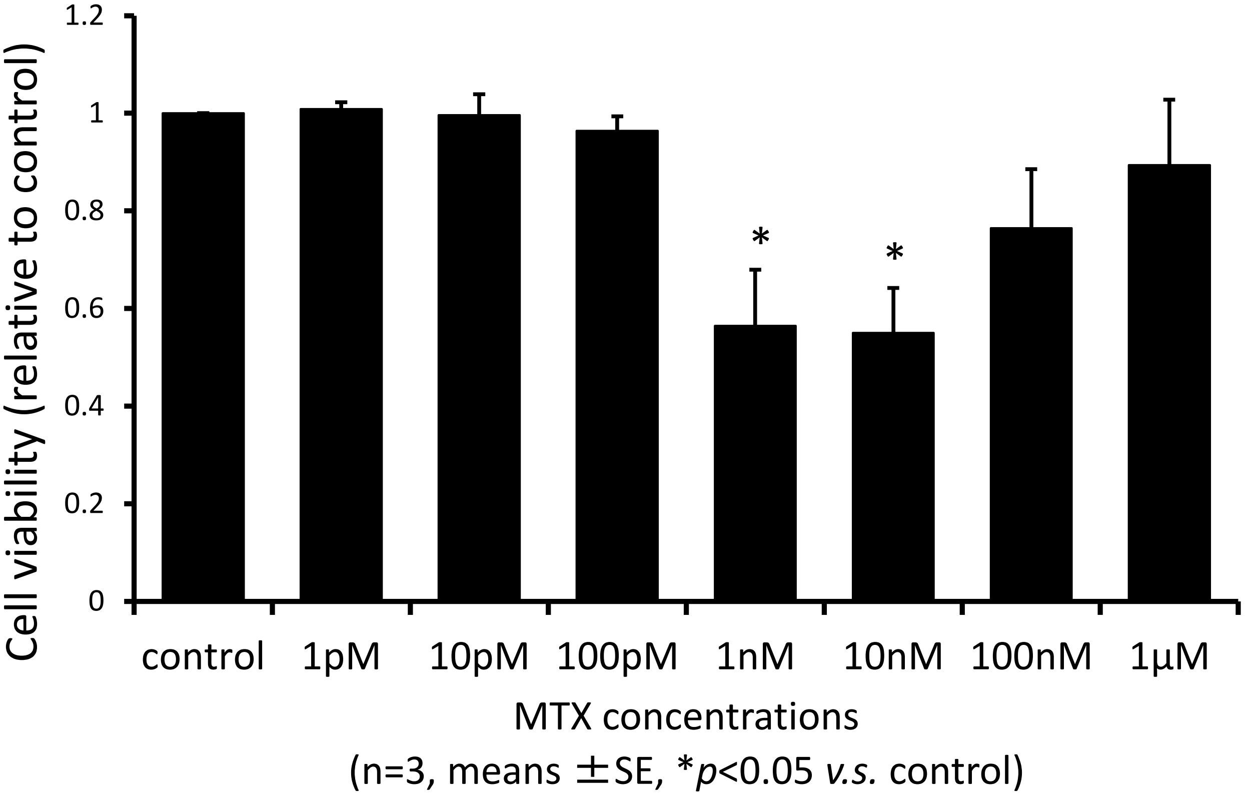

Additional file 4:

Cell viability of RA synovial fibroblasts was measured by WST-8 assay after 24 hrs' stimulation of MTX (1 pM to 1 μM).

1 and 10 nM of MTX significantly decreased the cell viability as shown in figure 1, while 1 to 100 pM of MTX didn't.
