# Supplementary material for: Methotrexate upregulates circadian transcriptional factors PAR bZIP to induce apoptosis on rheumatoid arthritis synovial fibroblasts
Source: Arthritis Res Ther. 2018 Mar 22;20:55. doi: 10.1186/s13075-018-1552-9 (PMC5863822; doi:10.1186/s13075-018-1552-9)
Supplement: Supplementary file 5 — The mRNA expression of circadian clock genes over time. mRNA expression of circadian clock genes measured at –4 h (before synchronization), 0 h (just before MTX stimulation), 24 h, 32 h, and 48 h. Controls and 10/100 nM of MTX showed almost the same expression rhythms, and MTX influenced their expression levels. (PDF 264 kb) [file 13075_2018_1552_MOESM5_ESM.pdf]

Additional file 5

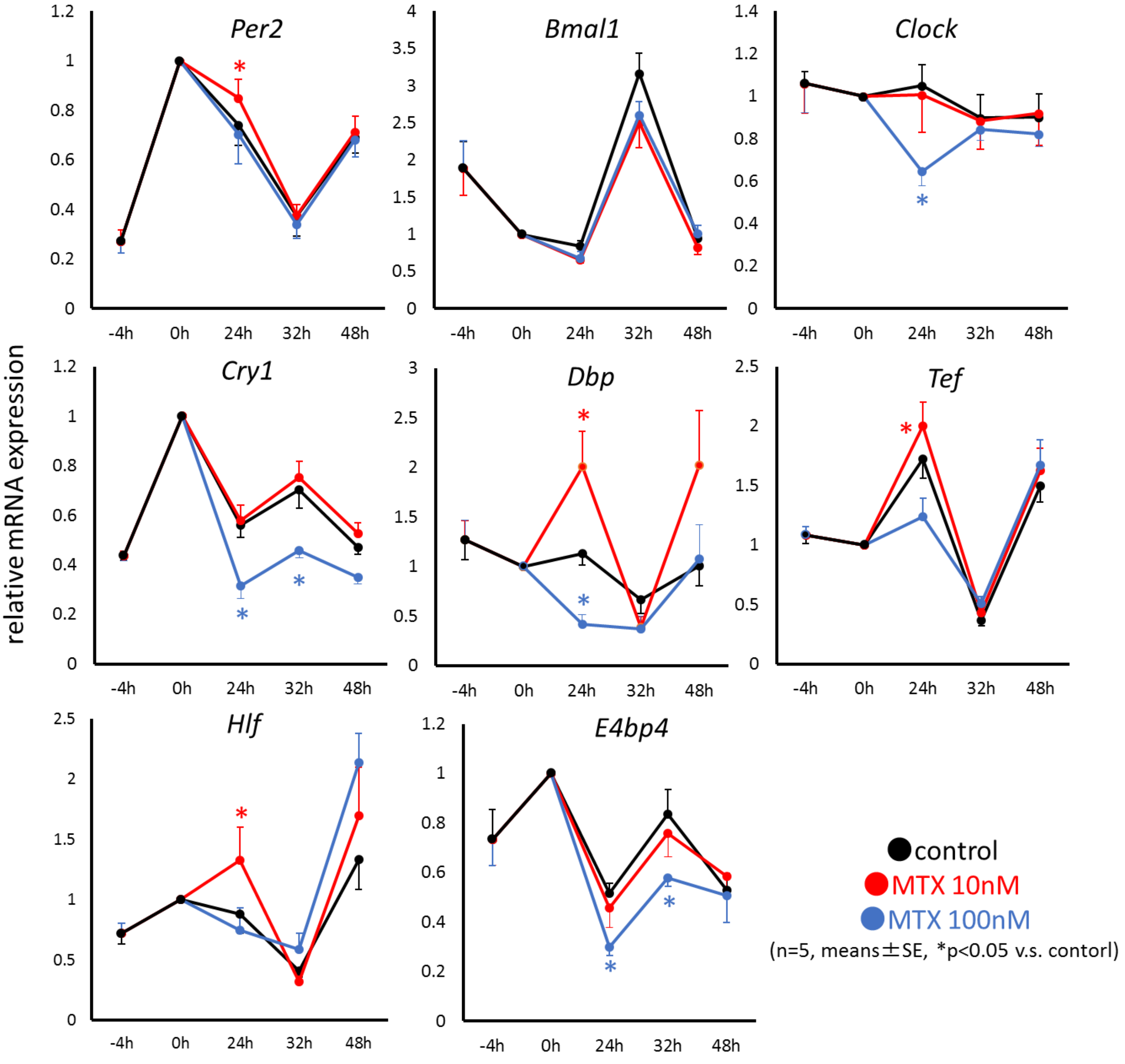


Additional file 5:
The mRNA expression of circadian clock genes were measured at -4h (before synchronization), 0h (just before MTX stimulation), 24h, 32h, and 48h.
We found that the controls and 10/100nM of MTX showed almost same expression rhythms, and MTX has influenced only on their expression levels.
