# Supplementary material for: Methotrexate upregulates circadian transcriptional factors PAR bZIP to induce apoptosis on rheumatoid arthritis synovial fibroblasts
Source: Arthritis Res Ther. 2018 Mar 22;20:55. doi: 10.1186/s13075-018-1552-9 (PMC5863822; doi:10.1186/s13075-018-1552-9)
Supplement: Supplementary file 6 — Two different transcriptional pathways by which MTX induces apoptosis to synovial fibroblasts: PAR bZIP–Per2 transcriptional pathway and PAR bZIP–Bik transcriptional pathway. We propose MTX induces apoptosis in synovial cells through activated binding of PAR bZIP to D-box in two different genes, Per2 and Bik, and these dual pathways work independently but synergistically. (PDF 206 kb) [file 13075_2018_1552_MOESM6_ESM.pdf]

Additional file 6

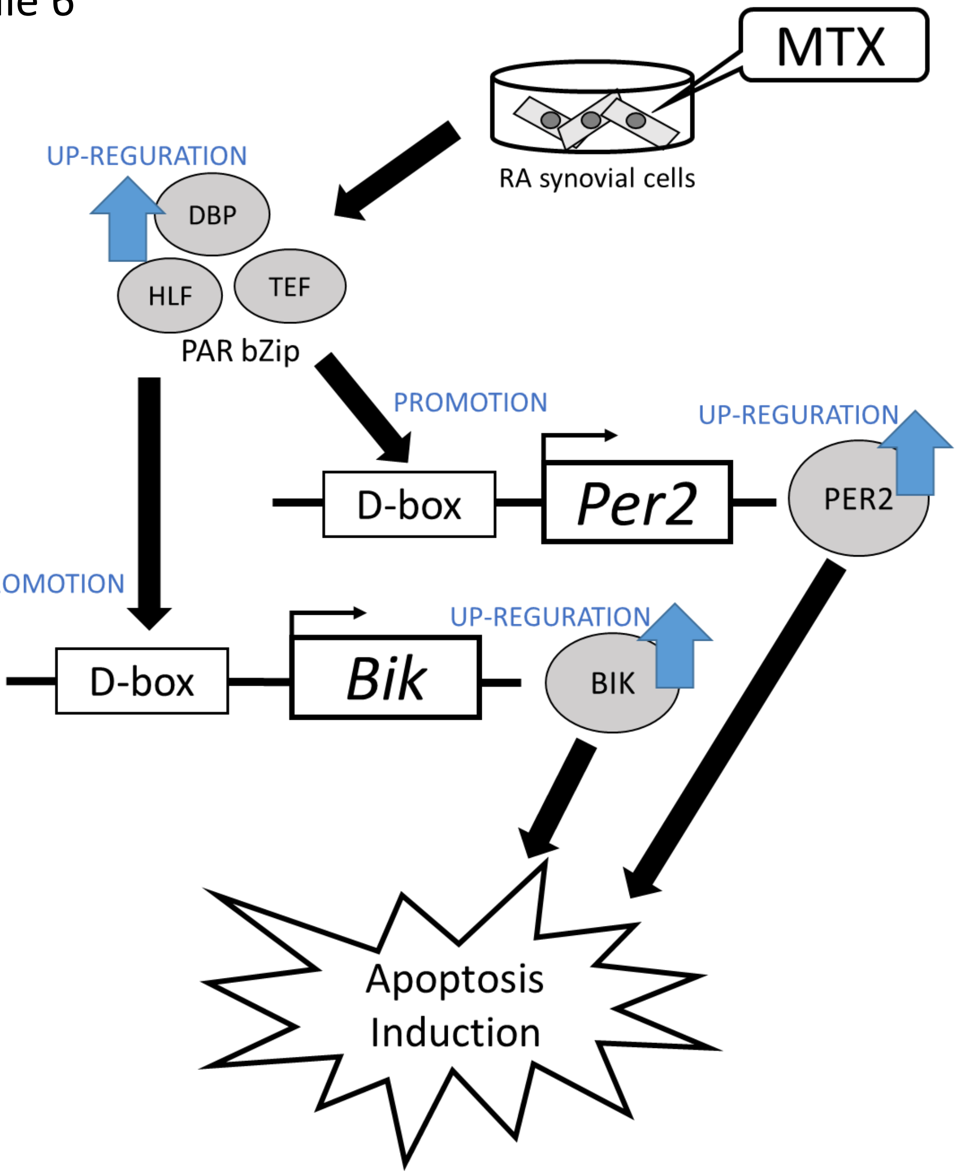

Additional file 6:

Two different transcriptional pathways that MTX induces apoptosis to synovial fibroblasts; PAR bZIP-Per2 transcriptional pathway and PAR bZIP-Bik transcriptional pathway.

We propose here that MTX induces apoptosis in synovial cells through activated binding of PAR bZIP to D-box in two different genes, *Per2* and *Bik*, and these dual pathways work independently, but synergistically.
